# Functional Independence and Self-Reported Depressive Symptoms 6–24 Months After Stroke: A Multicentre Cross-Sectional Study

**DOI:** 10.3390/healthcare14193253

**Published:** 2026-10-01

**Authors:** Marina Potašová, Eva Moraučíková, Katarína Zrubáková, Ivan Vargas, Peter Mačej

**Affiliations:** 1 Department of Physiotherapy, Catholic University in Ružomberok, 034-01 Ružomberok, Slovakia; marina.potasova@ku.sk; 2 Department of Nursing, Catholic University in Ružomberok, 034-01 Ružomberok, Slovakia; moraucikova@utb.cz; 3 Faculty of Humanities, Tomas Bata University in Zlín, 760 01 Zlín, Czech Republic; 4 Department of Psychology, University of Notre Dame, Notre Dame, IN 46556, USA; ivargas@nd.edu; 5 Rehabilitation Institute in Hrabyně, 747 67 Hrabyně, Czech Republic; peto.macej19@gmail.com

**Keywords:** post-stroke depression, functional independence, chronic stroke

## Abstract

**Highlights:**

**What are the main findings?**

- Functional independence and self-reported depressive symptoms showed a complex relationship in chronic stroke survivors.
- Social status emerged as an important factor associated with depressive symptom severity.

**What are the implications of the main findings?**

- Psychological well-being after stroke appears to be influenced by factors beyond the extent of physical disability.
- The findings support a comprehensive rehabilitation approach that addresses not only physical and functional deficits but also social rehabilitation and reintegration after stroke.

**Abstract:**

**Background:** Post-stroke depression (PSD) is one of the most common neuropsychological complications following stroke and is associated with greater functional disability and poorer functional outcomes. However, the relationship between self-reported depressive symptoms and functional independence remains insufficiently understood. The aim of this study was to examine the association between self-reported depressive symptoms and functional independence in patients in the chronic phase after stroke and examine selected sociodemographic and clinical factors associated with depressive symptom severity. **Methods:** A cross-sectional observational study was conducted in Slovakia and the Czech Republic. The sample consisted of 105 patients (64.75 ± 11.23 years) assessed 6–24 months after stroke. Functional independence was evaluated using the Functional Independence Measure and Functional Assessment Measure (FIM + FAM). Self-reported depressive symptoms were assessed using the Zung Self-Rating Depression Scale (SDS). Spearman correlation analysis and multiple linear regression were applied. **Results:** A significant positive correlation was identified between total FIM + FAM score and depressive symptom severity (ρ = 0.479, *p* < 0.001). Significant associations were also observed for motor (ρ = 0.475, *p* < 0.001), cognitive (ρ = 0.447, *p* < 0.001), and EADL domains (ρ = 0.544, *p* < 0.001), with the strongest relationship found in EADL. After adjustment for sociodemographic and clinical factors, total FIM + FAM was not independently associated with depressive symptom severity (B = 0.032, *p* = 0.079), and neither were its individual domains. Social status remained independently associated with depressive symptom severity, with patients living alone reporting lower depressive symptom scores. **Conclusions:** Functional independence was positively associated with depressive symptom severity in bivariate analyses but not after adjustment for sociodemographic and clinical factors. Social status remained independently associated with depressive symptom severity, highlighting the complex interplay between functional, social, and psychological factors after stroke.

## 1. Introduction

Stroke is one of the most serious global health challenges and represents a leading cause of mortality and long-term disability among adults [1]. The prevalence of individuals living with the consequences of stroke exceeds 80 million worldwide, and due to population ageing, its societal and healthcare burden is expected to increase further [2,3]. In Slovakia, 11,362 cases of stroke were recorded in 2022 [4].

In addition to motor and cognitive impairments, stroke survivors frequently experience neuropsychological complications that significantly affect the recovery process and functional independence. Among these, post-stroke depression (PSD) is the most common, affecting approximately 18–33% of patients after stroke. Despite its high prevalence and clinical relevance, post-stroke depression remains under-recognised and undertreated in routine clinical practice [5,6,7,8,9]. The etiology of PSD is multifactorial, involving both biological and psychosocial mechanisms, including structural brain damage, neurotransmitter dysregulation, and psychological responses to the loss of independence [10,11].

The relationship between depressive symptoms after stroke and functional independence constitutes an important area of research because depressive symptoms may reduce participation in rehabilitation, impair activities of daily living, decrease quality of life, and adversely affect long-term functional outcomes [8,10,12]. Previous studies have suggested an association between greater functional impairment and depressive symptoms after stroke. However, despite numerous studies examining the relationship between depressive symptoms and functional outcomes, the available evidence remains inconsistent, particularly with respect to the timing of depression assessment after stroke [13]. This is further supported by a recent systematic review and meta-analysis showing that both the prevalence and natural history of PSD vary over time after stroke, highlighting the importance of long-term follow-up in studies investigating depressive symptoms after stroke [14]. Consequently, evidence specifically addressing patients in the chronic phase after stroke remains limited, although psychological adaptation, social functioning, and awareness of disability may influence depressive symptom severity independently of physical impairment [15].

Despite the growing body of evidence on post-stroke depression, studies specifically investigating the relationship between depressive symptom severity and comprehensive functional independence in patients during the chronic phase after stroke remain limited. The primary aim of this study was to investigate the relationship between the severity of depressive symptoms and the level of functional independence in patients during the chronic phase after stroke. In addition, the associations between depressive symptom severity and selected sociodemographic factors.

## 2. Materials and Methods

### 2.1. Study Design

The study employed a cross-sectional observational design aimed at analysing the relationship between self-reported depressive symptoms and functional independence in patients in the chronic phase after stroke. The research was conducted between 27 September 2025 and 31 March 2026 in specialized rehabilitation and healthcare facilities in Slovakia and the Czech Republic. Data collection involved the following institutions: the National Rehabilitation Centre in Kováčová (*n* = 27), the Department of Internal Medicine and the Long-Term Care Unit of the Central Military Hospital SNP—FH in Ružomberok (*n* = 24), the Trojlístok Social Services Facility in Ružomberok (*n* = 20), and the Rehabilitation Institute Hrabyně, Czech Republic (*n* = 34). Participants were recruited consecutively from four participating inpatient centres. Following the initial medical examination at each centre, physicians screened patients according to the predefined eligibility criteria and referred potentially eligible individuals to the research team. Eligibility was further assessed by psychologists and speech and language therapists according to predefined criteria. At the facility without a psychologist or speech and language therapist (Trojlístok), eligibility was assessed by specialist physicians together with a physiotherapist from the research team. All participants were hospitalized at the time of assessment. The study assessment was performed within the first five days of hospitalization in order to minimize the potential influence of centre-specific rehabilitation and treatment procedures on functional and psychological outcomes. The assessment instruments were administered and scored by research team members who are qualified physiotherapists and nurses with extensive clinical experience. Standardized assessment and scoring procedures were applied across all centres. The Zung SDS was self-administered. In participants with visual difficulties, items were read aloud by the researcher without additional interpretation. Each researcher assessed participants at their respective clinical site. Therefore, formal inter-rater reliability testing was not performed. All procedures were conducted in accordance with the ethical principles of the Declaration of Helsinki and with the informed consent of the participants.

### 2.2. Participants

The study sample consisted of 105 patients (56 men and 49 women) with a mean age of 64.75 ± 11.23 years and a history of ischemic or haemorrhagic stroke diagnosed according to the International Classification of Diseases, 10th Revision (ICD-10). The upper limit of 24 months since stroke onset was established to ensure relative homogeneity of the sample with regard to long-term neuroplastic and psychosocial adaptation processes. All participants provided informed consent prior to enrolment in the study. The required sample size was estimated for a two-tailed correlation analysis, assuming a moderate effect size (r = 0.30), a significance level of α = 0.05, and a statistical power of 80%. The minimum required sample size was 84 participants. As the present study included 105 patients, the sample was considered adequate for the primary analysis. In addition, for the multivariable regression analysis with seven independent variables, assuming a medium effect size (Cohen’s f^2^ = 0.15), α = 0.05, and 80% power, the estimated minimum sample size was approximately 103 participants; thus, the available sample was also adequate for the adjusted regression model.

Patients meeting predefined eligibility criteria were included in the study. A confirmed diagnosis of ischemic or haemorrhagic stroke with a minimum post-stroke interval of six months was required, corresponding to the chronic phase characterized by relative neurological stability. Cognitive integrity was ensured by a score of ≥23 points on the Mini-Mental State Examination, indicating preserved comprehension and the ability to complete self-report measures reliably. Adequate communication abilities were also required and were defined by the absence of severe expressive or receptive aphasia that would preclude the administration of the assessment instruments. Furthermore, participants were required to have sufficient sensory capacity, including adequate visual and auditory functions, with or without compensation, allowing valid completion of the Zung Self-Rating Depression Scale. Psychiatric stability was ensured by excluding patients with severe psychiatric disorders, such as psychotic illnesses or advanced dementia, which could compromise the validity of self-reported depressive symptoms.

Patients with an acute intercurrent illness within the previous four weeks, a history of clinically significant depression treated prior to stroke onset, or current antidepressant therapy were also excluded from the study. Additional exclusion criteria included severe uncontrolled comorbidities, such as advanced heart failure (NYHA class IV), which may limit physical performance independently of neurological impairment, as well as severe apraxia or hemispatial neglect precluding the completion of the assessment procedures.

**Figure 1:**
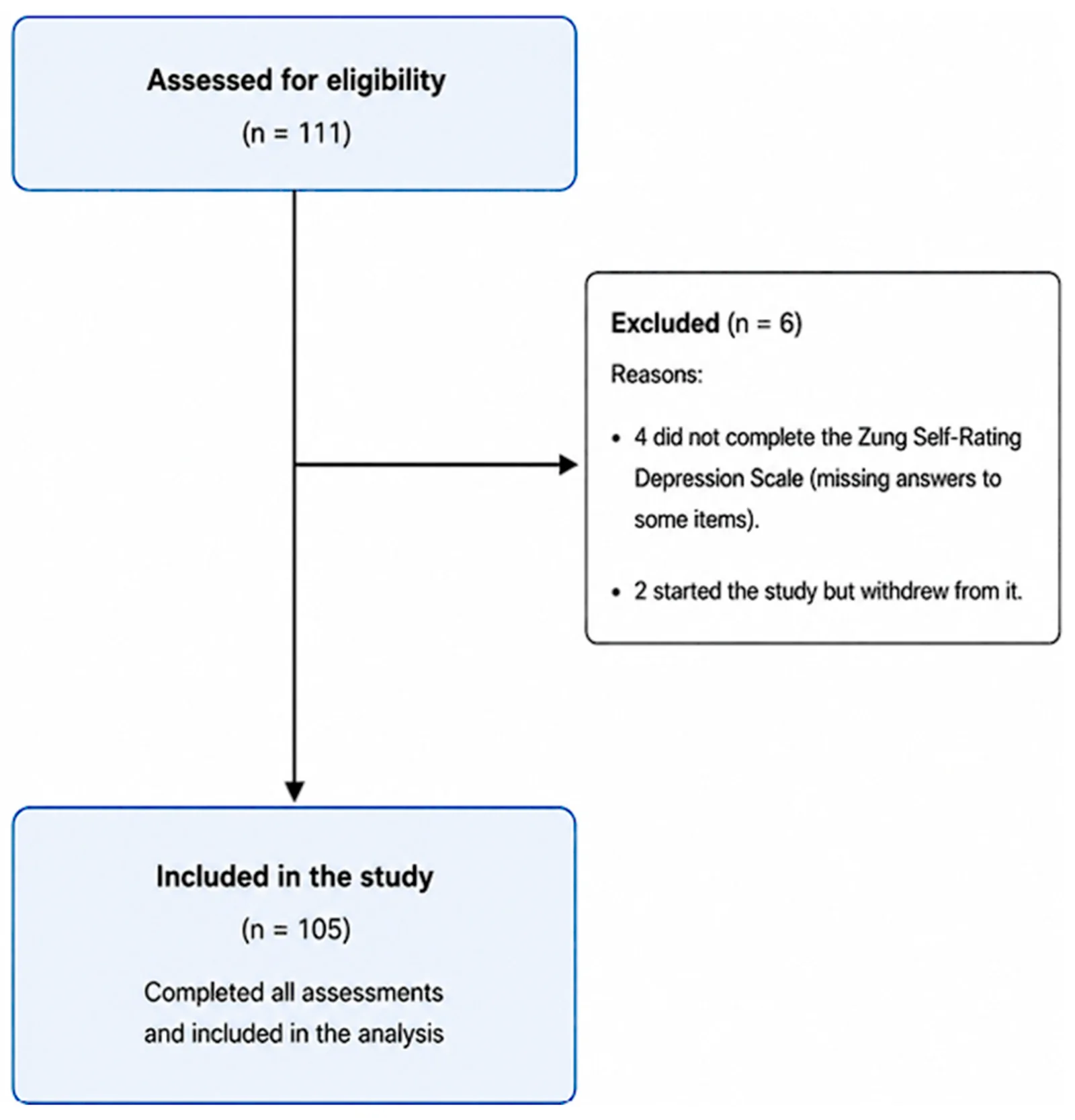
Flow diagram of participant recruitment and inclusion in the study.

Patients who clearly met the predefined exclusion criteria during the initial clinical screening were not referred to the research team and were therefore not included among the 111 patients assessed for study participation. Of the 111 patients assessed for eligibility, six were not included in the final analysis. Four participants returned incomplete Zung Self-Rating Depression Scale questionnaires and declined to complete the missing items. Therefore, their data could not be included in the analysis. Two additional participants initially provided consent but subsequently withdrew from the study during the assessment procedure without providing a reason. The final analytical sample therefore comprised 105 participants. The participant recruitment and selection process are presented in Figure 1.

### 2.3. Variables and Instruments

Several groups of variables were monitored and analysed using appropriate diagnostic instruments (Figure S1).

Among the sociodemographic variables, participants’ social status was assessed in order to evaluate the level of social support as a determinant of psychological well-being. Participants identified their current living situation by selecting one of three predefined categories: (a) living with a partner; (b) living alone (widowed or single); and (c) widowed or single individuals sharing a household with other family members. This variable was included in the analysis due to the presumed association between social isolation, or the quality of social support, and the development of depressive symptomatology, which has frequently been reported in clinical practice following neurological events.

Functional independence was assessed using the standardized Functional Independence Measure and Functional Assessment Measure (FIM + FAM), which demonstrates high validity and sensitivity in neurorehabilitation settings. The scale comprises 30 items, including the 18 core FIM items and an additional 12 FAM items focusing on cognitive and psychosocial aspects of disability [16,17,18]. To provide a more objective evaluation of adaptation to the home environment, the protocol was expanded by the Extended Activities of Daily Living (EADL) module, which includes six domains: meal preparation, laundry, household tasks, shopping, financial management, and participation in educational or work-related activities [19]. Scoring is based on a seven-point scale, where scores of 7 and 6 indicate independence, scores from 5 to 3 represent modified dependence, and scores of 2 and 1 reflect severe to complete dependence on assistance from others. The maximum cumulative score is 210 points, with separate analyses performed for the motor subscale (16–112 points) and the cognitive-psychosocial subscale (14–98 points) [19]. The instrument was translated into both Slovak and Czech languages.

The severity of depressive symptomatology was evaluated using the Zung Self-Rating Depression Scale (SDS). This validated psychometric instrument enables the assessment of affective, somatic, and psychomotor manifestations of depression symptoms. The questionnaire consists of 20 items. Participants rate the frequency of symptoms using a four-point Likert scale. Raw scores ranging from 20 to 80 are converted into an SDS index, which categorizes depressive symptoms into four levels of severity: normal range (<50), mild to moderate depression (50–59), moderate to severe depression (60–69), and severe depression (≥70) [20,21].

Overall clinical burden and mortality risk were assessed using the Charleson Comorbidity Index (CCI), a globally established scoring system for evaluating multimorbidity. The index assigns weighted scores ranging from 1 to 6 across 19 categories of chronic diseases according to their severity. The score also incorporates age adjustment, with one additional point assigned for each decade of life beyond the age of 50 years. The resulting cumulative score enables prediction of mortality risk and, within the context of the present study, was used to objectively assess the impact of comorbid conditions on psychological status [22,23].

### 2.4. Ethical Approval

The study was conducted at collaborating partner institutions in Slovakia and the Czech Republic. Ethical approval was granted by the Ethics Committee of the Central Military Hospital in Ružomberok (Reference No. ÚVN-18-54/2025, approval date: 23 September 2025). For the Czech participating center, Rehabilitation Institute Hrabyně, written institutional approval for conducting the study was provided by the Director of the Institute. All participants were thoroughly informed about the aims and methodological procedures of the study. Participation in the study was voluntary and was contingent upon the participants’ understanding of the provided information and the provision of written informed consent. The relevant supporting documentation is provided in the Supplementary Materials (Figure S2).

### 2.5. Statistical Analysis

Statistical analyses were performed using IBM SPSS Statistics Version 29.0.2.0(20). Data from all 105 participants were cleaned and the questionnaire instruments were scored prior to analysis. For the Zung Self-Rating Depression Scale, the ten positively worded items (items 2, 5, 6, 11, 12, 14, 16, 17, 18, and 20) were reverse-scored (5 − x) according to the standard scoring key. A raw score (range 20–80) was computed as the sum of all 20 items and converted to the SDS index using (raw score/80) × 100. All analyses used the SDS index. No questionnaire items were missing, and internal consistency in the present sample was Cronbach’s α = 0.621.

For the FIM + FAM, the motor (16 items) and cognitive (14 items) subscore were summed to yield the total score (range 30–210). The Extended Activities of Daily Living module (6 items, range 6–42) was scored separately and was not included in the total.

The normality of continuous variables was assessed using the Shapiro–Wilk test. As most were non-normally distributed, they are summarised as medians with interquartile ranges alongside means ± SD, and non-parametric methods were applied where appropriate. The association between functional independence and depressive symptom severity was examined using Spearman’s rank correlation.

Multivariable linear regression was used to examine factors independently associated with depressive symptom severity. The primary model specified the Zung SDS index as the dependent variable and total FIM + FAM as the principal exposure, adjusting for a priori-selected confounders—age, sex, social status, comorbidity burden, and time since stroke, chosen on clinical and theoretical grounds as variables plausibly influencing both functional independence and depressive symptoms. Secondary, exploratory models replaced total FIM + FAM with the motor, cognitive, and EADL subscore entered separately.

Because the age-adjusted Charlson Comorbidity Index incorporates age into its calculation, the primary model included the non-age-adjusted CCI together with chronological age to avoid duplication of age information. No problematic multicollinearity was observed (all VIFs ≤ 2.13). Alternative model specifications were examined in sensitivity analyses and yielded comparable substantive conclusions (Table S1).

Social status (three categories) was entered as dummy variables with “living with a partner” as the reference, and its overall contribution was assessed by a block F-change test.

Regression assumptions were examined: linearity and homoscedasticity from residual-versus-fitted plots, normality of residuals from normal probability plots, influential observations from Cook’s distance and standardised residuals, and multicollinearity from variance inflation factors (all < 2.7) (Figure S3).

Analyses were complete-case (*n* = 105). Ninety-five percent confidence intervals are reported for all coefficients. The association between total FIM + FAM and the Zung SDS index was the primary analysis. Domain-specific and single-variable analyses were exploratory. Statistical significance was set at α = 0.05.

## 3. Results

The following section presents the results of the study. Table 1 summarizes the baseline characteristics of the study population. Continuous variables are presented using mean ± standard deviation (SD) and median (Q1–Q3), while categorical variables are presented as number and percentage [*n* (%)].

The sample consisted of 105 patients with a mean age of 64.75 ± 11.23 years, with a slight predominance of males and ischemic stroke. Participants were assessed at a mean of 12.24 months after stroke onset, corresponding to the chronic phase of the disease. The cohort demonstrated a moderate level of functional impairment with relatively preserved motor and cognitive abilities. The mean Zung SDS index corresponded to mild depressive symptomatology.

The association between functional independence and the severity of depressive symptomatology is presented in Table 2.

The findings demonstrated a statistically significant relationship between the total FIM + FAM score and the corrected Zung Self- Rating Depression Index (Spearman’s rho = 0.479, *p* < 0.001). A moderate positive correlation was observed, indicating that higher levels of functional independence were associated with higher self-reported depressive symptom severity within the study cohort. Further analysis of the individual FIM + FAM domains revealed that depressive symptom severity was highly significantly associated (*p* < 0.001) with all investigated domains, although with slightly different strengths of association: Extended Activities of Daily Living (EADL), rho = 0.544; motor subscore (FIM), rho = 0.475; and cognitive subscore (FAM), rho = 0.447.

To further examine whether functional independence was independently associated with depressive symptom severity, a multiple linear regression analysis was performed Table 3). The Zung SDS index was entered as the dependent variable, with total FIM + FAM as the primary exposure and age, sex, social status, comorbidity burden, and time since stroke as covariates.

The overall regression model was statistically significant (N = 105; R = 0.652; R^2^ = 0.425; adjusted R^2^ = 0.384; F (7,97) = 10.26; *p* < 0.001; Durbin–Watson = 1.965). This is followed by an analysis of the primary and secondary models (Table 4 and Table 5).

In this adjusted model, higher total FIM + FAM showed a positive but non-significant association with depressive symptom severity (B = 0.032, 95% CI −0.004 to 0.067, *p* = 0.079). Thus, after adjustment for confounders, functional independence was not independently associated with depressive symptom severity, and the strong bivariate association (Spearman ρ = 0.479) was attenuated. Social status was the only factor significantly associated with depressive symptom severity. Patients living alone reported lower depressive symptoms than those living with a partner or family (B = −6.775, 95% CI −10.623 to −2.927, *p* < 0.001). Social status as a whole significantly improved the model (block F-change: F (2,97) = 6.35, *p* = 0.003) (Table S2).

The same adjusted model was applied separately with the motor, cognitive, and EADL sub scores as the exposure variables. The dependent variable was the Corrected Zung SDS Index.

Secondary models entering the motor, cognitive, and EADL subdomains separately showed the same pattern, all non-significant after adjustment (motor B = 0.050, *p* = 0.109; cognitive B = 0.052, *p* = 0.137; EADL B = 0.121, *p* = 0.163).

## 4. Discussion

The aim of the present study was to assess the relationship between self-reported depressive symptoms and functional independence in patients in the chronic phase after stroke and to examine selected sociodemographic and clinical factors associated with depressive symptom severity. The main finding was a significant positive bivariate association between the total FIM + FAM score and depressive symptom severity. However, after adjustment for sociodemographic and clinical factors, total FIM + FAM was not independently associated with depressive symptom severity. This finding suggests that the relationship between functional independence and depressive symptoms in the chronic phase after stroke cannot be explained by the level of functional impairment alone.

A similar pattern was observed across the individual functional domains. In bivariate analyses, the EADL, motor, and cognitive domains were all significantly positively associated with depressive symptom severity; however, after adjustment, none of these domains was independently associated with depressive symptomatology. These findings support the interpretation that the observed unadjusted associations may be influenced by additional social, clinical, or psychological factors. One possible hypothesis is that patients with greater functional independence may be more aware of the discrepancy between their premorbid and current condition. However, because self-awareness and patients’ perceptions of their own functional status were not directly assessed in this study, this interpretation should be considered exploratory. This interpretation is consistent with the heterogeneous findings reported in the available literature. Several studies have reported an association between depressive symptoms and poorer functional status [1,24,25], whereas other findings suggest that the severity of functional impairment may not be an independent factor associated with depressive symptomatology. For example, a three-year prospective study showed that the severity of functional deficits during the acute phase was not a clear predictor of subsequent depressive symptoms [26]. Similarly, the systematic review by Shewangizaw et al. showed that although most included studies identified an association between depression and poorer functional outcomes, some studies did not identify depression as an independent predictor of functional status [13]. Depressive symptoms are also common during the acute phase of stroke and may be associated with rehabilitation outcomes and quality of life [27]. These findings support a more complex view of the relationship between functional and psychological status after stroke. However, given the cross-sectional design of our study, the temporal direction of the observed associations cannot be established; depressive symptoms may be associated with functional status, but they may also influence self-perceived functioning, activity, or social participation.

An important finding of the adjusted analysis was the association with social status. Patients living alone reported lower levels of depressive symptoms than patients living with a partner and sharing a household. This unexpected finding should not be interpreted as indicating that living alone itself has a protective effect. One possible explanation may involve social and family roles, perceived dependence, and the nature of social support [28]. Patients living with a partner may perceive changes in their family and social roles more intensely and, in some cases, may feel dependent on others or perceive themselves as a burden to their relatives. This hypothesis is supported by studies in stroke populations in which greater self-perceived burden was associated with higher levels of depressive symptoms [29,30]. However, self-perceived burden, the quality of family relationships, and the nature of social support were not directly assessed in our study; therefore, this mechanism cannot be confirmed by our findings. Furthermore, the literature shows inconsistent associations between social support, marital status or living arrangements, and depressive symptoms after stroke [31]. Our finding should therefore be interpreted cautiously, taking into account the possibility of residual confounding, selection bias, centre-related differences, and limitations of self-reported assessment of depressive symptoms.

Sex was not independently associated with depressive symptom severity in the adjusted analysis. Although some studies have reported a higher prevalence of depressive symptoms among women after stroke [25,32], our findings did not confirm such an independent association.

From the perspective of future research, our findings support the need to evaluate the psychological status of patients after stroke within a broader context rather than solely in relation to physical or functional impairment. Future studies should consider social context, the quality of social support, changes in family roles, self-perceived burden, adaptive mechanisms, and self-awareness. Longitudinal studies may also help clarify the temporal relationships between functional recovery, social reintegration, and the development of depressive symptoms. These findings support the need for multidisciplinary rehabilitation that addresses not only motor and functional deficits but also the social and psychological reintegration of patients.

Several limitations should be considered. The cross-sectional design does not allow the temporal direction or causality of the observed associations to be established. Patient selection, particularly the exclusion of individuals receiving antidepressant treatment and those with substantial cognitive or communication impairment, limits the generalizability of the findings to the broader post-stroke population. Depressive symptoms were assessed using the self-report Zung SDS without a structured psychiatric interview. Some somatic SDS items may overlap with stroke-related sequelae, such as fatigue, sleep disturbances, appetite changes, or reduced psychomotor activity. The internal consistency of the SDS in our sample was modest (Cronbach’s α = 0.621), which should also be considered when interpreting the findings. Data on premorbid functional status, pre-stroke subclinical depressive symptoms, baseline stroke severity, and detailed lesion characteristics were unavailable. The 6–24-month post-stroke interval may also have contributed to heterogeneity. Differences between centres and countries cannot be fully excluded. In addition, education, socioeconomic status, rehabilitation intensity, and the quality of social support were not included in the analyses, which may have contributed to residual confounding.

## 5. Conclusions

The findings highlight the complex relationship between functional independence and self-reported depressive symptoms in patients during the chronic phase after stroke. Although functional independence was positively associated with depressive symptom severity in bivariate analyses, this association was not significant after adjustment for sociodemographic and clinical factors. Social status remained independently associated with depressive symptom severity. Given the cross-sectional design, causal conclusions cannot be drawn. Further longitudinal research is needed to clarify these relationships.

## Figures and Tables

**Table 1:** Baseline characteristics of the study population (*n* = 105).

Variable	Mean ± SD/*n* (%)	Median (Q1–Q3)
Age (years)	64.75 ± 11.23	65 (56–73)
Sex, male	56 (53.33)	-
Sex, female	49 (46.67)	-
Months after stroke	12.24 ± 7.76	7 (6–24)
Soc_1 (with a partner sharing household)	55 (52.4)	-
Soc_2 (living alone)	41 (39.0)	-
Soc_3 (single but sharing a household)	9 (8.6)	-
Ischemic stroke	79 (75.2)	-
Hemorrhagic stroke	26 (24.8)	-
Left hemisphere	65 (61.90)	-
Right hemisphere	40 (38.10)	-
CCI score	5.11 ± 1.9	5 (4–6)
Motor subscore (FIM)	67.53 ± 31.10	81 (30–90)
Cognitive subscore (FAM)	63.63 ± 26.09	74 (41–83)
EADL subscore	16.30 ± 10.18	15 (6–24)
Total FIM + FAM	131.16 ± 54.67	159 (68–170)
Zung SDS index	53.13 ± 8.60	53.75 (47.50–58.75)
Zung SDS index normal (<50)	37 (35.24)	-
Zung SDS index mild to moderate (50–59)	44 (41.90)	-
Zung SDS index moderate to severe (60–69)	23 (21.90)	-
Zung SDS index severe (≥70)	1 (0.95)	-

**Table 2:** Correlation between overall functional independence and the severity of depressive symptomatology.

Correlation			Zung_SDS_Index
Spearman’s rho	Cognitive subscore	Correlation Coefficient	0.447 **
		Sig. (2-tailed)	<0.001
		N	105
	Motor subscore	Correlation Coefficient	0.475 **
		Sig. (2-tailed)	<0.001
		N	105
	EADL subscore	Correlation Coefficient	0.544 **
		Sig. (2-tailed)	<0.001
		N	105
	Total FIM + FAM	Correlation Coefficient	0.479 **
		Sig. (2-tailed)	<0.001
		N	105

** Correlation is significant at the 0.01 level (2-tailed).

**Table 3:** A multiple linear regression analysis.

**Model Summary ^b^**					
**R**	**R Square**	**Adjusted R Square**	**Std. Error of the Estimate**	**Durbin-Watson**	
0.652 ^a^	0.425	0.384	6.778	1.965	
**ANOVA ^c^**					
	**Sum of Squares**	**df**	**Mean Square**	**F**	**Sig.**
Regression	3298.181	7	471.169	10.256	<0.001 ^d^
Residual	4456.352	97	45.942		
Total	7754.533	104			

a. Independent variables: (Constant), months since stroke, sex (0 = male, 1 = female), soc_3, non-age-adjusted, soc_2, Total FIM + FAM, age; b. Dependent Variable: Corrected Zung SDS Index; c. Dependent Variable: Corrected Zung SDS Index; d. Independent variables: (Constant), months since stroke, sex (0 = male, 1 = female), soc_3, non-age-adjusted, soc_2, Total FIM + FAM, age.

**Table 4:** Primary Regression Model.

Independent Variables	B	Std. Error	β	t	*p*	95% CI Lower	95% CI for Upper	Tolerance	VIF
(Constant)	52.409	6.464		8.108	<0.001	39.581	65.238		
Total FIM + FAM	0.032	0.018	0.201	1.775	0.079	−0.004	0.067	0.469	2.130
Months since stroke	−0.194	0.106	−0.175	−1.829	0.071	−0.404	0.017	0.658	1.520
Age	0.055	0.075	0.072	0.736	0.463	−0.094	0.205	0.627	1.596
Sex (0 = male, 1 = female)	0.864	1.368	0.050	0.632	0.529	−1.851	3.578	0.951	1.052
Non-age-adjusted CCI	−0.738	0.556	−0.110	−1.327	0.187	−1.842	0.366	0.875	1.143
soc_2	−6.775	1.939	−0.385	−3.494	< 0.001	−10.623	−2.927	0.496	2.016
soc_3	−1.564	2.502	−0.051	−0.625	0.533	−6.529	3.402	0.904	1.106

Dependent Variable: Corrected Zung SDS Index.

**Table 5:** Secondary Regression Models.

Secondary Model	B (Domain)	*p*	95% CI	VIF	R^2^
Motor Subscore	0.050	0.109	−0.011 to 0.112	2.10	0.424
Cognitive Subscore	0.052	0.137	−0.017 to 0.120	1.83	0.422
EADL Subscore	0.121	0.163	−0.050 to 0.293	1.73	0.420

## Data Availability

The de-identified data supporting the conclusions of this article are available from the corresponding author upon reasonable request, subject to ethical and grant-related restrictions. Statistical code is not available because the analyses were performed by an external statistical expert.

## Supplementary Materials

The following supporting information can be downloaded at: https://www.mdpi.com/article/10.3390/healthcare14193253/s1, Figure S1: Patient Assessment Form Used in the Study EN/CZ/SV versions; Figure S2: Ethical approval; Approval of the Research Request—Hrabyně Rehabilitation Institute, Czech Republic and Informed consent form used in the study ENJ/SK/CZ; Figure S3: Diagnostic Assessment of Regression Model Assumptions; Table S1: Sensitivity Analysis of Regression Model Specification; Table S2: Overall Contribution of Social Status to the Regression Model.

## Abbreviations

The following abbreviations are used in this manuscript:

ACA	Anterior Cerebral Artery
CCI	Charlson Comorbidity Index
EADL	Extended Activities of Daily Living
FAC	Functional Ambulation Categories
FAM	Functional Assessment Measure
FIM	Functional Independence Measure
IBM	International Business Machines Corporation
ICD	International Classification of Diseases
MCA	Middle Cerebral Artery
MZ SR	Ministry of Health of the Slovak Republic
NCZI	National Health Information Center
NYHA	New York Heart Association
PCA	Posterior Cerebral Artery
PSD	Post-stroke depression
SDS	Self-Rating Depression Scale
SPSS	Statistical Package for the Social Sciences
