# Supplementary material for: Functional Independence and Self-Reported Depressive Symptoms 6–24 Months After Stroke: A Multicentre Cross-Sectional Study
Source: Healthcare (Basel). 2026 Oct 1;14(19):3253. doi: 10.3390/healthcare14193253 (PMC13665235; doi:10.3390/healthcare14193253)
Supplement: Supplementary file 1 [file healthcare-14-03253-s001.zip › Figure S1_30.9..pdf]

Figure S1: Patient Assessment Form Used in the Study EN/CZ/SV versions

Patient Assessment after Stroke

The following information should be completed by healthcare professionals based on clinical examination and/or medical records.

Initials:...................................... Age:........................ sex: **M / F** High................. Weight................

Months since stroke onset: ....

**Social Status**<br>1. Married or living with a partner in the same household<br>2. Widowed/single living alone<br>3. Widowed/single living with family	**Stroke Type**<br>• Ischemic stroke<br>• Hemorrhagic stroke<br>**Affected side:**<br>• Dominant extremities<br>• Non-dominant extremities
**Vascular Territory Involved (MRI, CT)**<br>1. ACA (Arteria cerebri anterior)<br>2. MCA (Arteria cerebri media)<br>3. PCA (Arteria cerebri posterior)<br>4. LAC (Lacunar Stroke)<br>5. Cerebelerna artéria<br>6. A.basilaris	**Rehabilitation**<br>• Inpatient rehabilitation<br>• Outpatient rehabilitation<br>• Home-based rehabilitation<br>• No rehabilitation
**Treatment**<br>1. Pharmacological<br>2. Surgical	**Comorbidities:**

Functional Ambulation Category (FAC)

**0 – Non-functional ambulation**	**1 – Dependent ambulation, level II**	**2 – Dependent ambulation, level I**	**3 – Dependent for supervision**	**4 – Independent ambulation on level surfaces**	**5 – Independent ambulation**
Unable to walk or requires assistance from more than one person.	Requires continuous physical contact from one person to prevent falling.	Requires intermittent or light physical contact from one person.	Requires supervision only, without physical contact.	Requires supervision or assistance only when negotiating obstacles. Independent on level surfaces.	Independent walking outdoors and in the community. Assistive devices may be used.

Functional Independence Measure and Functional Assessment Measure (FIM+FAM)

**Fuction**	**Score**	**Assign scores according to the following criteria:**
Eating		**7 – Complete Independence**<br>The patient performs the activity independently.<br>**6 – Modified Independence**<br>The patient performs the entire activity independently but requires assistive devices, additional time, or a modified environment (e.g., grab bars, raised toilet seat).<br>**5 – Supervision**<br>The patient performs the activity independently but requires supervision, verbal cues, or assistance with setup or preparation of the environment.<br>**4 – Minimal Assistance**<br>The patient performs most of the activity independently.
Swallowing		
Grooming		
Bathing		
Upper body dressing		
Lower body dressing		
Toileting		
Bladder assitance		
Bowel assitance		
Bed, chair, wheelchair transfer		
Toilet transfer		
Tub, shower transfer		
Car transfer		

<table>
<tr><td>Locomotion Walking / wheelchair</td><td></td><td rowspan="24">Light physical contact (e.g., support for balance) or minor verbal assistance is required.
3 – Moderate Assistance
The patient requires obvious physical assistance from another person but still performs more than half of the activity independently.
2 – Maximal Assistance
The patient requires substantial physical assistance from one person and performs less than half of the activity independently.
1 – Total Assistance
The patient is unable to perform any part of the activity independently and requires complete physical assistance from one or more persons.</td></tr>
<tr><td>Stairs</td><td></td></tr>
<tr><td>Community mobility</td><td></td></tr>
<tr><td>Comprehension</td><td></td></tr>
<tr><td>Expression</td><td></td></tr>
<tr><td>Reading</td><td></td></tr>
<tr><td>Writing</td><td></td></tr>
<tr><td>Speech Intelligibility</td><td></td></tr>
<tr><td>Social interaction</td><td></td></tr>
<tr><td>Emotional status</td><td></td></tr>
<tr><td>Adjustment to limitations</td><td></td></tr>
<tr><td>Leisure Activities</td><td></td></tr>
<tr><td>Problem solving</td><td></td></tr>
<tr><td>Memory</td><td></td></tr>
<tr><td>Orientation</td><td></td></tr>
<tr><td>Contretacion</td><td></td></tr>
<tr><td>Safety awareness</td><td></td></tr>
<tr><td>Extended Activities of Daily Living (EADL)</td><td></td></tr>
<tr><td>Meal preparation</td><td></td></tr>
<tr><td>Laundry</td><td></td></tr>
<tr><td>Housework</td><td></td></tr>
<tr><td>Shopping</td><td></td></tr>
<tr><td>Financial management</td><td></td></tr>
<tr><td>Work/education</td><td></td></tr>
</table>

Zung Self-Rating Depression Scale (ZUNG)

Instructions:

Please read each statement and decide how much of the time the statement describes how you have been feeling during the past several days.

		A little of the time	Some of the time	Good part of the time	Most of the time
1	I feel down-hearted and blue	1	2	3	4
2	Morning is when I feel the best	4	3	2	1
3	I have crying spells or feel like it	1	2	3	4
4	I have trouble sleeping at night	1	2	3	4
5	I eat as much as I used to	4	3	2	1
6	I still enjoy sex	4	3	2	1
7	I notice that I am losing weight	1	2	3	4
8	I have trouble with constipation	1	2	3	4
9	My heart beats faster than usual	1	2	3	4
10	I get tired for no reason	1	2	3	4
11	My mind is as clear as it used to be	4	3	2	1
12	I find it easy to do the things I used to	4	3	2	1
13	I am restless and can't keep still	1	2	3	4
14	I feel hopeful about the future	4	3	2	1
15	I am more irritable than usual	1	2	3	4
16	I find it easy to make decisions	4	3	2	1
17	I feel that I am useful and needed	4	3	2	1
18	My life is pretty full	4	3	2	1
19	I feel that others would be better off if I were dead	1	2	3	4
20	I still enjoy the things I used to do.	4	3	2	1

Czech version

Patient Assessment after Stroke

Hodnocení pacienta po cévní mozkové příhodě

Následující údaje vyplní zdravotničtí pracovníci na základě klinického vyšetření a/nebo zdravotnické dokumentace.

Iniciály: .... Věk: .... Pohlaví: **M / Ž** Výška: ....
Hmotnost: .... Počet měsíců od vzniku cévní mozkové příhody: ....

**Sociální status**<br>1. Ženatý/vdaná nebo žijící s partnerem ve společné domácnosti<br>2. Vdovec/vdova / svobodný(á), žijící sám/sama<br>3. Vdovec/vdova / svobodný(á), žijící s rodinou	**Typ cévní mozkové příhody**<br>• Ischemická cévní mozková příhoda<br>• Hemoragická cévní mozková příhoda<br>**Postižená strana:**<br>• Dominantní končetiny<br>• Nedominantní končetiny
**Postižené cévní povodí (MRI, CT)**<br>1. ACA (Arteria cerebri anterior)<br>2. MCA (Arteria cerebri media)<br>3. PCA (Arteria cerebri posterior)<br>4. LAC (Lacunar Stroke)<br>5. Cerebelerna artéria<br>6. A.basilaris	**Rehabilitace**<br>• Lůžková rehabilitace<br>• Ambulantní rehabilitace<br>• Domácí rehabilitace<br>• Bez rehabilitace
**Léčba**<br>1. Farmakologická<br>2. Chirurgická	**Komorbidity:**

Functional Ambulation Category (FAC)

**0 – Nefunkční chůze**	**1 – Závislá chůze, úroveň II**	**2 – Závislá chůze, úroveň I**	**3 – Závislost na dohledu**	**4 – Samostatná chůze po rovném povrchu**	**5 – Samostatná chůze**
Není schopen/schopna chůze nebo vyžaduje pomoc více než jedné osoby.	Vyžaduje nepřetržitý fyzický kontakt jedné osoby k zabránění pádu.	Vyžaduje občasný nebo lehký fyzický kontakt jedné osoby.	Vyžaduje pouze dohled, bez fyzického kontaktu.	Vyžaduje dohled nebo pomoc pouze při překonávání překážek. Po rovném povrchu je chůze samostatná.	Samostatná chůze venku a v komunitním prostředí. Mohou být používány kompenzační pomůcky.

Functional Independence Measure and Functional Assessment Measure (FIM+FAM)

**Funkce**	**Skóre**
Stravování	
Příjem potravy	
Polykání	
Osobní hygiena	
Koupání	
Oblékání horní části těla	
Oblékání dolní části těla	
Kontrola močového měchýře	
Kontrola vyprazdňování stolice	
Lůžko, židle, invalidní vozík	
Toaleta	
Vana, sprcha	
Automobil	
Chůze / mobilita na invalidním vozíku	
Schody	

Přidělte skóre podle následujících kritérií:

7 – Úplná soběstačnost
Pacient provádí aktivitu samostatně.
6 – Modifikovaná soběstačnost
Pacient provádí celou aktivitu samostatně, ale potřebuje kompenzační pomůcky, více času nebo upravené prostředí (např. madla, zvýšené toaletní sedátko).
5 – Dohled
Pacient provádí aktivitu samostatně, ale vyžaduje dohled, slovní pokyny nebo pomoc s přípravou či úpravou prostředí.
4 – Minimální asistence
Pacient provádí většinu aktivity samostatně. Je vyžadován lehký fyzický kontakt (např. podpora rovnováhy) nebo drobná slovní pomoc.

<table>
<tr><td>Mobilita v komunitním prostředí</td><td></td><td rowspan="22">3 – Střední asistence
Pacient vyžaduje zřetelnou fyzickou pomoc jiné osoby, ale stále provádí více než polovinu aktivity samostatně.
2 – Maximální asistence
Pacient vyžaduje značnou fyzickou pomoc jedné osoby a samostatně provádí méně než polovinu aktivity.
1 – Úplná asistence
Pacient není schopen provést žádnou část aktivity samostatně a vyžaduje úplnou fyzickou pomoc jedné nebo více osob.</td></tr>
<tr><td>Porozumění</td><td></td></tr>
<tr><td>Vyjadrovanie</td><td></td></tr>
<tr><td>Čtení</td><td></td></tr>
<tr><td>Psaní</td><td></td></tr>
<tr><td>Komunikace</td><td></td></tr>
<tr><td>Sociální interakce</td><td></td></tr>
<tr><td>Emoční stav</td><td></td></tr>
<tr><td>Přizpůsobení se omezením</td><td></td></tr>
<tr><td>Využívání volného času</td><td></td></tr>
<tr><td>Řešení problémů</td><td></td></tr>
<tr><td>Paměť</td><td></td></tr>
<tr><td>Orientace</td><td></td></tr>
<tr><td>Koncentrácia</td><td></td></tr>
<tr><td>Uvědomování si bezpečnosti</td><td></td></tr>
<tr><td>Rozšířené aktivity denního života (EADL)</td><td></td></tr>
<tr><td>Příprava jídla</td><td></td></tr>
<tr><td>Praní prádla</td><td></td></tr>
<tr><td>Domácí práce</td><td></td></tr>
<tr><td>Nakupování</td><td></td></tr>
<tr><td>Správa financí</td><td></td></tr>
<tr><td>Práce/vzdělávání</td><td></td></tr>
</table>

Sebeposuzovací stupnice deprese podle Zhunga

	Nikdy nebo zřídka	Někdy	Dosti často	Velmi často nebo stále
1. Jsem smutný, skleslý , sklíčený	☐ 1	☐ 2	☐ 3	☐ 4
2. Nejlépe se cítím ráno	☐ 4	☐ 3	☐ 2	☐ 1
3. Bývá mi do pláče nebo dokonce pláču	☐ 1	☐ 2	☐ 3	☐ 4
4. V noci špatně spím	☐ 1	☐ 2	☐ 3	☐ 4
5. Mám stejnou chuť k jídlu a jím jako dříve	☐ 4	☐ 3	☐ 2	☐ 1
6. Sexuální život a myšlenky na něj mi činí potěšení jako dříve	☐ 4	☐ 3	☐ 2	☐ 1
7. Všiml/a jsem si, že ubývám na váze	☐ 1	☐ 2	☐ 3	☐ 4
8. Mám potíže se zácpou	☐ 1	☐ 2	☐ 3	☐ 4
9. Mívám rychlý tep nebo bušení srdce	☐ 1	☐ 2	☐ 3	☐ 4
10. Jsem unavený/á bez zjevné příčiny	☐ 1	☐ 2	☐ 3	☐ 4
11. Moje myšlení je jasné jako vždy dříve	☐ 4	☐ 3	☐ 2	☐ 1
12. Všechny obvyklé činnosti zvládám bez problémů	☐ 4	☐ 3	☐ 2	☐ 1
13. Cítím nepokoj a nevydržím v klidu	☐ 1	☐ 2	☐ 3	☐ 4
14. Do budoucna nahlížím s nadějí	☐ 4	☐ 3	☐ 2	☐ 1
15. Bývám podrážděný/á	☐ 1	☐ 2	☐ 3	☐ 4
16. Není pro mě problém rozhodnout se	☐ 4	☐ 3	☐ 2	☐ 1
17. Cítím, že jsem užitečný/á a potřebný/á	☐ 4	☐ 3	☐ 2	☐ 1
18. Cítím, že žiji naplno	☐ 4	☐ 3	☐ 2	☐ 1
19. Mám pocit, že pro ostatní by bylo lépe, kdybych tu nebyl/a	☐ 1	☐ 2	☐ 3	☐ 4
20. Těší mě stejné věci tak jako dříve	☐ 4	☐ 3	☐ 2	☐ 1

Slovak version

Vyšetrenie pacienta po mozgovej príhode

Uvedené údaje o pacientovi vyplní personál na základe vyšetrenia resp. zdravotnej dokumentácie.

Iniciály:...................................... Vek:....................... Pohlavie: **M / Ž** Výška................. Váha................

Počet mesiacov od vzniku NCMP: ....

**Sociálny status (odpoveď zakrúžkujte)**<br>1. manželstvo alebo spolužitie v jednej domácnosti<br>2. ovdovený / slobodný žijúci sám<br>3. ovdovený /slobodný žijúci s rodinou	**Typ NCMP (odpoveď zakrúžkujte)**<br>4. ischemická / hemoragická<br>**Postihnuté:**<br>5. dominantné končatiny/ nedominantné končatiny;
**Poškodenie cievy (nález MR alebo CT, od lekára alebo z chorobopis) - (odpoveď zakrúžkujte)**<br>7. ACA (Arteria cerebri anterior)<br>8. MCA (Arteria cerebri media)<br>9. PCA (Arteria cerebri posterior)<br>10. LAC (Lacunar Stroke)<br>11. Cerebelerna artéria<br>12. A.basilaris	**Rehabilitácia (odpoveď zakrúžkujte)**<br>1. Absolvovaný liečebný pobyt v ústavnom zariadení (rehabilitačné oddelenie, kúpele, NRC Kováčová a pod.)<br>2. Ambulantná liečba v rámci bydliska<br>3. Liečba v domácom prostredí v rámci ADOS<br>4. Neabsolvoval žiadnu rehabilitáciu
**Liečba (odpoveď zakrúžkujte):**<br>1. Farmakologická<br>2. Operačná	**Pacientove pridružené ochorenia (vypíšte):**

FAC škála (zakrúžkujte číslo, ktoré charakterizuje chôdzu pacienta)

**0 – neschopný** chôdze, alebo chôdza s asistenciou viac ako 1 osoby	**1 -** Chôdza závislá od **fyzického kontaktu 1 osoby,** aby sa predišlo pádu.	**2- občasný,** ľahký fyzický **kontakt**.	**3 - len dohľad** bez fyzického kontaktu.	**4- dohľad** alebo asistenciu **len pri** prekonávaní **prekážok**. Po rovine samostatný**.**	**5- Samostatná** chôdza v teréne. Môže používať pomôcky.

FIM+FAM – prideľte body (**1 až 7**) za schopnosť pacienta zvládnuť jednotlivé činnosti podľa inštrukcií uvedených v tabuľke.

**Činnosti**		**Body**	**Definované skórovanie (udeľovanie bodov za jednotlivé činnosti)**
1	Jedenie		**Body prideľte podľa nasledovných kritérií:**<br><br>**7- Pacient je samostatný**<br><br>**6- Modifikovaná samostatnosť (vykonáva celú činnosť samostatne, ale potrebuje pomôcky, dlhšie mu to trvá alebo vyžaduje modifikované prostredie (madlá, zvýšená toaleta).**
2	Prehĺtanie		
3	Osobná hygiena		
4	Kúpanie		
5	Obliekanie hornej polovice tela		
6	Obliekanie dolnej polovice tela		
7	Použitie WC		
8	Kontrola močenia		
9	Kontrola stolice		
10	Lôžko, stolička, vozík		

11	Toaleta	
12	Vaňa, sprchový kút	
13	Auto	
14	Chôdza, mobilita na vozíku	
15	Schody	
16	Pohyb v komunite	
17	Porozumenie	
18	Vyjadrovanie	
19	Čítanie	
20	Písanie	
21	Zrozumiteľnosť reči	
22	Sociálna interakcia	
23	Emočný stav	
24	Prispôsobenie limitáciám	
25	Využitie voľného času	
26	Riešenie problémov	
27	Pamäť	
28	Orientácia	
29	Koncentrácia	
30	Bezpečnostné uvedomenie	
**Rozšírené aktivity denného života**		
1	Príprava stravy	
2	Pranie	
3	Starostlivosť o domácnosť	
4	Nakupovanie	
5	Financie	
6	Práca/vzdelávanie	

5- Dohľad - činnosť vykoná sám, ale potrebuje dohľad, verbálne pokyny alebo nastavenie/prípravu prostredia.

4 – Minimálna asistencia - väčšinu vykonáva činnosti sám. Potrebuje ľahký fyzický kontakt (napr. podpora rovnováhy) alebo miernu verbálnu pomoc.

3 - stredná asistencia - Potrebuje zjavnú fyzickú pomoc od inej osoby, ale stále vykonáva viac ako polovicu činností.

2 – maximálna asistencia- Potrebuje značnú fyzickú pomoc 1 osoby a s ňou vykonáva menej ako polovicu činnosti sám.

1 - Úplná závislosť - nevykonáva žiadnu časť činnosti. Potrebuje úplnú fyzickú pomoc od jednej alebo viacerých osôb.

Sbahodnotiaca stupnica depresívných syptomov podľa Zhunga

Otázka č.		Nikdy alebo zriedka	Niekedy	Dosť často	Veľmi často nebo stále
1	Som smutný, skleslý, skľúčený	1	2	3	4
2	Najlepšie sa cítim ráno	4	3	2	1
3	Býva mi do plaču, alebo dokonca aj plačem	1	2	3	4
4	V noci zle spím	1	2	3	4
5	Mám rovnakú chuť k jedlu a jedávam rovnako ako predtým	4	3	2	1
6	Sexuálny život a myšlienky naň mi robia rovnakú radosť ako predtým	4	3	2	1
7	Všimol /a som si úbytok na váhy	1	2	3	4
8	Mám ťažkosti so zápchou	1	2	3	4
9	Mávam rýchly tep alebo búšenie srdca	1	2	3	4
10	Som unavený/á bez zjavnej príčiny	1	2	3	4
11	Moje myslenie je jasné ako predtým	4	3	2	1
12	Všetky bežné činnosti zvládam bez problémov	4	3	2	1
13	Cítim nepokoj a nevydržím v kľude	1	2	3	4
14	Na budúcnosť sa dívam s nádejou	4	3	2	1
15	Bývam podráždený/á	1	2	3	4
16	Nemám problém rozhodnúť sa	4	3	2	1
17	Cítim, že som užitočný/á a potrebný/á	4	3	2	1
18	Cítim, že žijem naplno	4	3	2	1
19	Mám pocit, že pre ostatných by bolo lepšie, keby som tu nebol/a	1	2	3	4
20	Tešia ma veci rovnako ako predtým	4	3	2	1
