# Supplementary material for: Functional Independence and Self-Reported Depressive Symptoms 6–24 Months After Stroke: A Multicentre Cross-Sectional Study
Source: Healthcare (Basel). 2026 Oct 1;14(19):3253. doi: 10.3390/healthcare14193253 (PMC13665235; doi:10.3390/healthcare14193253)
Supplement: Supplementary file 1 [file healthcare-14-03253-s001.zip › Figure S2_30.9..pdf]

Figure S2. Ethical approval; Approval of the Research Request – Hrabyně Rehabilitation Institute, Czech Republic and Informed consent form used in the study ENJ/SK/CZ

Version

ETHICAL APPROVAL

Ústredná vojenská nemocnica SNP Ružomberok
- fakultná nemocnica
Gen. Miloša Vesela 21, 034 26 Ružomberok

Žiadosť o povolenie prieskumu v Ústrednej vojenskej nemocnici SNP Ružomberok – fakultnej nemocnici

Žiadateľ:

Priezvisko, meno, titul: MARINA POTAŠOVÁ Mgr. PhD

Dátum narodenia: 10.08.1985 Mobil: 0948 399 635 Email: marina.potasova@ku.sk

Adresa: KOŠŤANY NAD TURCOM 353, 038 41 KOŠŤ. N. TURCOM

Pracovisko: FZ KU, KATEDRA FYZIOTERAPIE

Vzdelávacia ustanovizeň: .... Forma štúdia: ☐ denná ☐ externá

Téma záverečnej práce:* IMPACT OF INTERPLAY BETWEEN INDIPENDENCE AND DEPRESION IN PATIENTS AFTER STROKE

Spôsob zberu dát - prieskum/výskum/dotazník/test/iná analýza (vzor priložiť ako prílohu)

Termín zberu dát: od: 1.9.2025 do: 31.8.2026

Pracovisko dotazníkového prieskumu: NEUROLOGICKÉ AMBULANCIE A ODDELENIA

Prezentácia dát:

- ☐ bakalárska práca
- ☐ diplomová práca
- ☐ špecializačná / atestačná práca
- ☐ rigorózna práca
- ☐ doktorandská práca
- ☒ publikačná činnosť

Poučenie:

Žiadateľ sa zaväzuje, že zachová mlčanlivosť o skutočnostiach zistených v súvislosti s uskutočňovaným výskumom a zbieraním dát. Použité dotazníky a poznatky z nich získané budú anonymné. Po spracovaní dát, žiadateľ predloží získané výsledky Etickej komisii, ktorá výskum/dotazníkový prieskum povolila. Prezentácia výsledkov, s uvedením Ústrednej vojenskej nemocnice SNP Ružomberok – fakultnej nemocnice ako miestom konania prieskumu, je možná iba s jej súhlasom a získané výsledky budú použité výhradne v záverečnej práci žiadateľa/ky.

Dátum: 14.07.2025 Podpis žiadateľa: Potašová

*možnosť priloženia krátkej anotácie, prípadne osnovy

Stanovisko referátu vedy a výskumu a následné predloženie Etickej komisie:

Ústredná vojenská nemocnica SNP Ružomberok
Fakultná nemocnica 5
Dátum prijatia: 27. AUG 2025
Číslo záznamu: 54 Číslo spisu: ÚVN-18-54/2025
Počet listov: Spracovateľ:
Počet príloh: 1/

Číslo žiadosti: ÚVN-18-54/2025

☒ Súhlasím, a následne predkladám na Etickú komisiu
☐ Nesúhlasím

Dňa: 27. AUG 2025

Oddelenie vedy, výskumu a vzdelávania
Mgr. Lenka Palcová, PhD., MPH
vedúca oddelenia

Referát vedy, výskumu, vzdelávania

Stanovisko Etickej komisie:

☒ Súhlasím ☐ Nesúhlasím

Dňa: 18. SEP 2025

Ústredná vojenská nemocnica SNP Ružomberok
Fakultná nemocnica
ETICKÁ KOMISIA 1

Predseda Etickej komisie

Podpis riaditeľa nemocnice:

Dňa: 23. SEP 2025

MUDr. Marian Križko, PhD., MPH, MHA
Riaditeľ nemocnice

Riaditeľ nemocnice

Držiteľ certifikátu
Systému manažérstva kvality ISO 9001
Telefón: +421 44 438 2900
Email: palcoval@uvn.sk
Web: www.uvn.sk

SGS

Bankové spojenie Štátna pokladnica Bratislava
Číslo účtu IBAN SK 84 81800000 00700017 7393
IČO: 319 364 15
DIČ: 2020590187
IČ DPH: SK2020590187

APPROVAL OF THE RESEARCH REQUEST – HRABYNĚ REHABILITATION INSTITUTE CZ/EN

Rehabilitační ústav Hrabyně, 747 67 Hrabyně, Česká republika

Mgr. Marina Potašová, PhD.
Katolická univerzita v Ružomberku
Fakulta zdravotnictví
Nám. A. Hlinku 48
034 01 Ružomberok
Slovenská republika
Tel.: +421 948 399 635

Věc: Žádost o souhlas s realizací výzkumu

Vážený pane řediteli,
na základě dlouholeté mezinárodní spolupráce se na Vás obracím se žádostí o povolení realizace výzkumu na rehabilitačním oddělení Vašeho ústavu.
Výzkum bude probíhat v období od 1. října 2025 do 31. března 2026 a jeho cílem je získání podkladů pro publikaci článku ve vědeckém časopise. Sběrem dat je pověřen kolega PhDr. Peter Mačej, PhD., který ve Vašem zařízení působí jako fyzioterapeut.
Informace o zpracování údajů:

- **Účel:** Získané údaje budou použity výhradně jako podklad pro publikaci vědeckého článku.
- **Anonymita:** Výzkum je plně anonymní. Výsledky budou interpretovány souhrnně a nebudou obsahovat žádné osobní údaje účastníků, které by mohly vést k jejich identifikaci.
- **Průběh:** Výzkum bude organizován tak, aby nenarušoval běžný chod zařízení ani harmonogram pacientů či zaměstnanců.

V případě potřeby doplnění dalších informací mě, prosím, neváhejte kontaktovat.
Předem Vám děkuji za vstřícnost.

Přílohy:
1. Souhlas Etické komise v Ružomberku
2. Informovaný souhlas pacientů
3. Vyšetřovací formulář – FIM+FAM a Zungova sebeposuzovací škála deprese

V Ružomberku dne 29. 09. 2025

Mgr. Marina Potašová, PhD.
Hlavní řešitel projektu

Vyjádření ředitele: * souhlasím / ~~nesouhlasím s realizací výzkumu.~~

Podpis ředitele a razítko zařízení

FAKULTNÍ NEMOCNICE OSTRAVA
REHABILITAČNÍ ÚSTAV HRABYNĚ A CHUCHELNÁ
Hrabyně č. 204, 747 67 Hrabyně
IČO: 00843989

* nehodící se škrtněte

Rehabilitation Institute Hrabyně, 747 67 Hrabyně, Czech Republic

Mgr. Marina Potašová, PhD.
Catholic University in Ružomberok
Faculty of Health Sciences
Nám. A. Hlinku 48
034 01 Ružomberok
Slovak Republic
Tel.: +421 948 399 635

Subject: Request for Approval to Conduct a Research Study

Dear Director,
based on our long-standing international cooperation, I am writing to request your approval to conduct a research study at the rehabilitation department of your institute.
The study will be conducted from 1 October 2025 to 31 March 2026 and aims to collect data for the preparation of a scientific article. Data collection will be carried out by our colleague PhDr. Peter Mačej, PhD., who works at your institution as a physiotherapist.
Information on data processing:

- **Purpose:** The collected data will be used exclusively as a basis for the preparation and publication of a scientific article.
- **Anonymity:** The study is fully anonymous. The results will be reported in aggregate form and will not contain any personal data that could lead to the identification of individual participants.
- **Procedure:** The study will be organized so as not to interfere with the routine operation of the institution or the schedules of patients and staff.

Should you require any additional information or documentation, please do not hesitate to contact me.
Thank you in advance for your cooperation and consideration.

Attachments:

1. Approval of the Ethics Committee in Ružomberok
2. Patient informed consent form
3. Assessment form – FIM+FAM and the Zung Self-Rating Depression Scale

Ružomberok, 29 September 2025

Mgr. Marina Potašová, PhD.
Principal Investigator

Director's decision: * I approve / ~~I do not approve the conduct of the study.~~

Director's signature

FAKULTNÍ NEMOCNICE OSTRAVA
REHABILITAČNÍ ÚSTAV HRABYNĚ A CHUCHELNÁ
Hrabyně č. 204, 747 67 Hrabyně
IČO: 00843989

* Delete as appropriate

INFORMED CONSENT

Dear Sir / Madam,

I hereby request your consent to participate in the research conducted within the project Nanovic Institute for European Studies, University of Notre Dame, NI-08-04KU titled **Impact of the interplay between indipendence and depresion in patients after stroke**, which is carried out under the auspices of the Catholic University in Ružomerok. The research is conducted in accordance with the Universal Declaration of Human Rights, the General Data Protection Regulation (GDPR), and Act No. 18/2018 Coll. on the protection of personal data and on amendments to certain acts, as amended, and other generally binding regulations (such as the Helsinki Declaration adopted at the 18th World Health Assembly in 1964, as amended (Fortaleza, Brazil, 2013); the Act on Health Care and Services Related to the Provision of Health Care No. 576/2004 Coll.; and the Declaration on Human Rights and Biomedicine No. 96/2001).

Name and surname of the submitter and principal investigator of the project: Mgr. Marina Potašová, PhD

Signature: ....

Name and surname of the person who provided the instruction:

Signature: ....

I declare and confirm with my handwritten signature below that I voluntarily agree to participate in the above-mentioned project, and that I have had the opportunity to properly and within sufficiently long time consider all relevant information about the research, ask all essential questions related to participation in the research, and that I have received clear and comprehensible answers to my comments. I have been informed of the right to refuse participation in the research project or to withdraw my consent at any time without any repercussions.

Name and surname:

Signature: ....

Place: ………………………………… Date: ………………………

INFORMOVANÝ SÚHLAS

Vážená pani / Vážený pán,

týmto Vás žiadam o súhlas s účasťou na výskume realizovanom v rámci projektu Nanovic Institute for European Studies, University of Notre Dame, NI-08-04KU s názvom **Vplyv vzájomného pôsobenia nezávislosti a depresie u pacientov po cievnej mozgovej príhode**, ktorý sa uskutočňuje pod záštitou Katolíckej univerzity v Ružomberku. Výskum sa realizuje v súlade so Všeobecnou deklaráciou ľudských práv, Všeobecným nariadením o ochrane údajov (GDPR), zákonom č. 18/2018 Z. z. o ochrane osobných údajov a o zmene a doplnení niektorých zákonov v znení neskorších predpisov a ďalšími všeobecne záväznými právnymi predpismi (napr. Helsinskou deklaráciou prijatou na 18. Svetovom zdravotníckom zhromaždení v roku 1964 v znení neskorších zmien (Fortaleza, Brazília, 2013); zákonom č. 576/2004 Z. z. o zdravotnej starostlivosti, službách súvisiacich s poskytovaním zdravotnej starostlivosti a o zmene a doplnení niektorých zákonov; a Dohovorom o ľudských právach a biomedicíne č. 96/2001 Z. z.).

Meno a priezvisko predkladateľa a hlavného riešiteľa projektu:
Mgr. Marina Potašová, PhD.

Podpis: ....

Meno a priezvisko osoby, ktorá poskytla poučenie:

Podpis: ....

Vyhlasujem a svojím vlastnoručným podpisom nižšie potvrdzujem, že dobrovoľne súhlasím s účasťou na vyššie uvedenom projekte a že som mal/a možnosť riadne a počas dostatočne dlhého času zvážiť všetky relevantné informácie o výskume, položiť všetky podstatné otázky súvisiace s účasťou na výskume a že som na svoje otázky a pripomienky dostal/a jasné a zrozumiteľné odpovede. Bol/a som informovaný/á o práve odmietnuť účasť vo výskumnom projekte alebo svoj súhlas kedykoľvek odvolať bez akýchkoľvek následkov.

Meno a priezvisko: ....

Podpis: ....

Miesto: .... **Dátum:** ....

INFORMOVANÝ SOUHLAS

Vážená paní / Vážený pane,

tímto Vás žádám o souhlas s účastí ve výzkumu realizovaném v rámci projektu Nanovic Institute for European Studies, University of Notre Dame, NI-08-04KU s názvem **Vliv vzájemného působení nezávislosti a deprese u pacientů po cévní mozkové příhodě**, který je realizován pod záštitou Katolické univerzity v Ružomberku. Výzkum je prováděn v souladu se Všeobecnou deklarací lidských práv, obecným nařízením o ochraně osobních údajů (GDPR), zákonem č. 18/2018 Z. z. o ochraně osobních údajů a o změně a doplnění některých zákonů ve znění pozdějších předpisů a dalšími obecně závaznými právními předpisy (např. Helsinskou deklarací přijatou na 18. Světovém zdravotnickém shromáždění v roce 1964 ve znění pozdějších změn (Fortaleza, Brazílie, 2013); zákonem č. 576/2004 Z. z. o zdravotní péči, službách souvisejících s poskytováním zdravotní péče a o změně a doplnění některých zákonů; a Úmluvou o lidských právech a biomedicíně č. 96/2001 Z. z.).

Jméno a příjmení předkladatele a hlavního řešitele projektu:
Mgr. Marina Potašová, PhD.

Podpis: ....

Jméno a příjmení osoby, která poskytla poučení:

Podpis: ....

Prohlašuji a svým vlastnoručním podpisem níže potvrzuji, že dobrovolně souhlasím s účastí ve výše uvedeném projektu a že jsem měl/a možnost řádně a po dostatečně dlouhou dobu zvážit všechny relevantní informace o výzkumu, položit všechny podstatné otázky související s účastí ve výzkumu a že jsem na své otázky a připomínky obdržel/a jasné a srozumitelné odpovědi. Byl/a jsem informován/a o právu odmítnout účast ve výzkumném projektu nebo svůj souhlas kdykoli odvolat bez jakýchkoli následků.

Jméno a příjmení: ....

Podpis: ....

Místo: ……………………………… **Datum:** ………………………………
