# Supplementary material for: Functional Independence and Self-Reported Depressive Symptoms 6–24 Months After Stroke: A Multicentre Cross-Sectional Study
Source: Healthcare (Basel). 2026 Oct 1;14(19):3253. doi: 10.3390/healthcare14193253 (PMC13665235; doi:10.3390/healthcare14193253)

Figure S3: Diagnostic Assessment of Regression Model Assumptions

Residuals (Python): Shapiro–Wilk p = 0.039 (mild deviation), Breusch–Pagan p = 0.023 (mild heteroscedasticity).

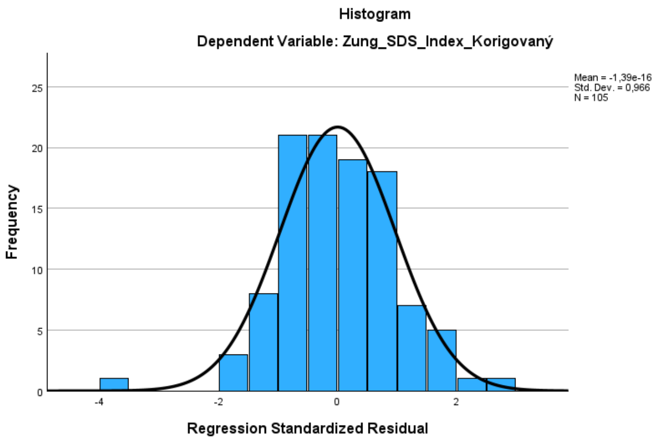

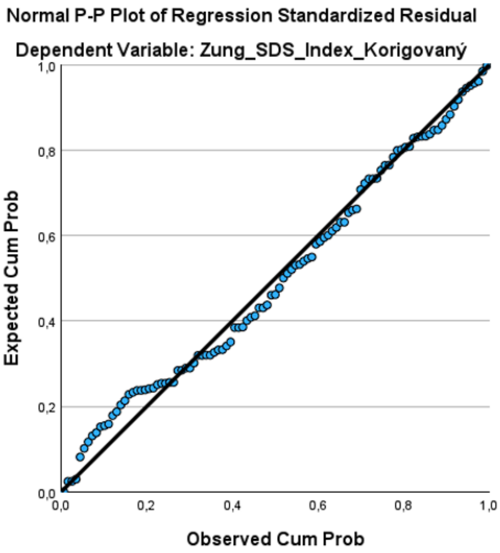

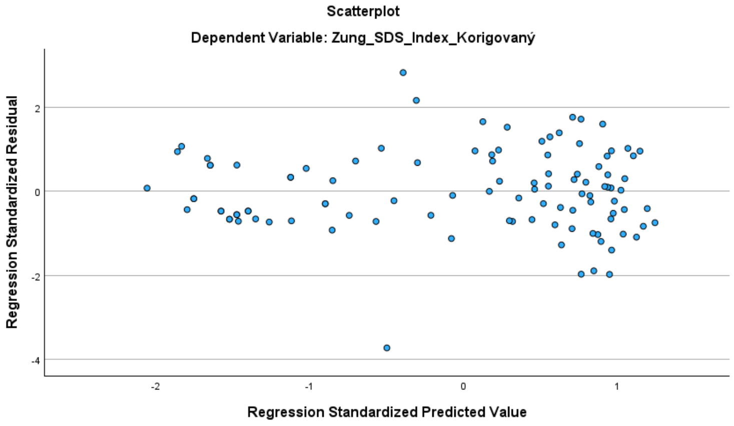

Supplement: Supplementary file 1 [file healthcare-14-03253-s001.zip › Figure S3_30.9..pdf]
