# Supplementary material for: Functional Independence and Self-Reported Depressive Symptoms 6–24 Months After Stroke: A Multicentre Cross-Sectional Study
Source: Healthcare (Basel). 2026 Oct 1;14(19):3253. doi: 10.3390/healthcare14193253 (PMC13665235; doi:10.3390/healthcare14193253)
Supplement: Supplementary file 1 [file healthcare-14-03253-s001.zip › Table S1_30.9..pdf]

Table S1: Sensitivity Analysis of Regression Model Specification

Model	Age B (p), VIF	CCI B (p), VIF	FIM+FAM B (p)	R^2
Original model (age-adjusted CCI + age)	0.156 (0.110), 2.67	−0.980 (0.079), 2.44	0.030 (0.092)	0.425
**Variant A (non-age-adjusted CCI + age)**	0.055 (0.463), 1.60	−0.738 (0.187), 1.14	0.032 (0.079)	0.417
Variant B (age-adjusted CCI, without age)	—	−0.411 (0.341), 1.45	0.027 (0.127)	0.410

To address the potential duplication of age information arising from the simultaneous inclusion of chronological age and the age-adjusted Charlson Comorbidity Index (CCI), two alternative model specifications were examined. Variant A included chronological age together with the non-age-adjusted CCI, whereas Variant B included the age-adjusted CCI without chronological age. These models were compared with the original model containing both chronological age and the age-adjusted CCI.

Variant A was selected for the primary analysis. Separating chronological age from comorbidity burden reduced the VIF for age from 2.67 to 1.60 and for CCI from 2.44 to 1.14, while allowing both variables to remain independently interpretable. The substantive conclusions were unchanged across all three model specifications: total FIM+FAM was not independently associated with depressive symptom severity after adjustment, whereas soc_2 remained the only stable significant predictor. In Variant A, neither chronological age ($p = 0.463$) nor the non-age-adjusted CCI ($p = 0.187$) was independently associated with depressive symptom severity.
