# Supplementary material for: Functional Independence and Self-Reported Depressive Symptoms 6–24 Months After Stroke: A Multicentre Cross-Sectional Study
Source: Healthcare (Basel). 2026 Oct 1;14(19):3253. doi: 10.3390/healthcare14193253 (PMC13665235; doi:10.3390/healthcare14193253)
Supplement: Supplementary file 1 [file healthcare-14-03253-s001.zip › Table S2_30.9..pdf]

Table S2: Overall Contribution of Social Status to the Regression Model

Model Summary

Model	R	R Square	Adjusted R Square	Std. Error of the Estimate	R Square Change	F Change	df1	df2	Sig. F Change
1	0.592[a]	0.350	0.317	7.135	0.350	10.668	5	99	< 0.001
2	0.652[b]	0.425	0.384	6.778	0.075	6.345	2	97	0.003

a. Predictors: (Constant), Total FIM+FAM, Sex (0 = male, 1 = female), CCI score, Months since stroke, Age.

b. Predictors: (Constant), Total FIM+FAM, Sex (0 = male, 1 = female), CCI score, Months since stroke, Age, soc_3, soc_2.
